# Computed tomographic cervical myelography in horses: Technique and findings in 51 clinical cases

**DOI:** 10.1111/jvim.15848

**Published:** 2020-07-24

**Authors:** Sarah L. Gough, Jonathan D. C. Anderson, Jonathon J Dixon

**Affiliations:** ^1^ Rainbow Equine Hospital North Yorkshire United Kingdom

**Keywords:** arthritis, articular process joint, compression, equine, wobbler

## Abstract

**Background:**

Three‐dimensional computed tomographic (CT) evaluation of the cervical vertebral column enables more accurate identification of osseous and soft tissue lesions than traditional latero‐lateral radiography. However, examination of the complete cervical vertebral column has been limited by horse size, preventing evaluation of the caudal cervical vertebrae.

**Objectives:**

To describe a technique to enable CT myelography of the complete cervical spine and describe the findings in 51 horses.

**Animals:**

Records of 51 horses presented for evaluation of cervical vertebral lesions.

**Methods:**

A retrospective review of clinical records from all horses presented for CT myelography to further investigate possible cervical vertebral lesions was performed. A description of a novel approach to CT myelography in horses and retrospective review of the findings in clinical cases has been included.

**Results:**

Degenerative joint disease was identified at 1 or more dorsal articular process joint in 50/51 horses, of which 44/51 had a site of grade 2 or greater. Spinal cord compression was observed on CT myelography in 31/51 horses, whereas attenuation of the dorsal contrast column was identified radiographically in 11/50 horses. Thirty‐three horses showed narrowing or obliteration of the intervertebral foramina at 1 or more site and osteochondral fragments were seen in 11/51 horses.

**Conclusions and Clinical Importance:**

Computed tomography myelography is relatively safe and an easily performed technique with the correct equipment, enabling evaluation of the cervical vertebral structures of horses in all planes and volumetrically. It is possible that lesion extent might be underestimated with this diagnostic modality, hence interpretation should be complimented with flexed and extended views radiographically.

**Abbreviations**
APJ: articular process joint
CSF: cerebrospinal fluid
CT: computed tomography
CVSM: cervical vertebral stenotic myelopathy
DCC: dorsal contrast column
DJD: degenerative joint disease
IVR: intervertebral ratio
MRI: magnetic resonance imaging
SCC: spinal cord compression

## 1 INTRODUCTION

Historically, evaluation of the cervical vertebral column for evidence of vertebral canal stenosis, spinal cord compression (SCC), and degenerative joint disease (DJD) of the articular process joints (APJs) in equids has relied on radiographs and radiographic myelography.^1^, ^2^, ^3^, ^4^ Survey radiography has a sensitivity of 42 and 63% for vertebral canal stenosis identified with intravertebral ratio (IVR) and DJD, respectively, when compared with necropsy, and radiographic myelography (judged by 50% reduction in the dorsal contrast column [DCC]) has a sensitivity of 71 and 43% when compared with necropsy.^5^ Although radiographic myelography enables assessment of SCC by measurement of contrast attenuation in flexed, neutral and extended positions, and measurement of both intervertebral and intravertebral ratios, three‐dimensional examination is not possible.^1^, ^6^, ^7^ Misdiagnosis of compressive lesions with survey radiography alone occurs in 30%‐60% of cases compared with radiographic myelography,^8^ and 61% of cases compared with necropsy,^5^ highlighting the importance of myelography for evaluation of compressive lesions. Similarly, accurate evaluation of the impact of DJD of the APJ on SCC is limited.^5^, ^6^, ^8^ However, in a multicenter study, radiographic myelography had a 68% correlation with necropsy findings,^5^ indicating that radiographic myelography frequently underestimates presence of cervical SCC. Computed tomography (CT) and CT myelography are advocated for tomographic evaluation of the cervical vertebral column and identification of SCC.^9^, ^10^, ^11^ CT and CT myelography confers multiple advantages including avoidance of superimposition of adjacent structures enabling accurate identification of lesions circumferentially, multiplanar image reconstruction and examination for SCC in any image plane. This might facilitate diagnosis of articular process impingement on spinal nerves as well as SCC.

The use of a specifically designed and adapted CT gantry to enable CT myelography under general anesthesia and the findings of CT myelography in both cadavers and live horses has been reported.^11^, ^12^ Compressive spinal cord lesions confirmed histologically were more likely to be accurately detected using CT over radiographic techniques, with fewer false‐positive diagnoses.^12^ This study describes in detail a technique for CT and CT myelography of the cervical and cranial thoracic vertebral canal in clinical cases, and the CT and CT myelographic findings in 51 horses in which cervical SCC was considered a differential diagnosis.

## 2 MATERIALS AND METHODS OF THE NOVEL CT MYELOGRAPHY TECHNIQUE

A thorough neurological examination was performed in all cases, and was considered indicated for neurolocalization and identification of abnormalities that might influence the safety of general anesthesia. Standing survey radiographs were performed if a fracture or subluxation of the cervical vertebrae was suspected. Before induction of general anesthesia, an area measuring 20 cm × 20 cm caudally to the occipital protuberance and equilaterally from the midline was clipped. A catheter was aseptically placed in the right jugular vein and sutured in position. Horses received 0.02 mg/kg acepromazine (Acesedate, Jurox UK, West Sussex) intramuscularly (IM) in combination with 0.06 mg/kg romifidine (Sedivet, Boehringer Ingelheim Vetmedica, Inc, St Joseph, Missouri) IV as anesthetic premedication. Additional medication administered included 1.1 mg/kg flunixin meglumine (Flunixin, Norbrook Laboratories LTD, Northern Ireland, UK) IV and 0.1 mg/kg dexamethasone (Colvasone, Norbrook Laboratories LTD) IV. Anesthesia was induced with 2.5 mg/kg ketamine (Anestekin, Dechra Pharmaceuticals PLC, Bladel, the Netherlands) in combination with 0.05 mg/kg diazepam (Diazepam, Hameln Pharmaceuticals LTD, Gloucester, UK) IV and maintained with a triple drip (500 mL [50 g] guaifenesin [Myorelax, Dechra Pharmaceuticals PLC, Northwich, UK], 15 mL [1.5 g] ketamine [Anestekin, Dechra Pharmaceuticals PLC], 3 mL [30 mg] romifidine [Sedivet, Boehringer Ingleheim Vetmedica, Inc]) at an initial rate of body weight (in kg) plus 50 mL per hour. Additional boluses of ketamine (Anestekin, Dechra Pharmaceuticals PLC) were administered at 0.2 to 0.8 mg/kg where necessary in animals that developed a lighter plane of anesthesia, or 0.5 mg/kg of thiopental (Thiopental Sodium 500 mg, Archimedes Pharma UK LTD, Galashiels, UK) if movement was noted. An endotracheal tube was passed on some but not all horses with clinician preference dictating its use.

**FIGURE 1:**
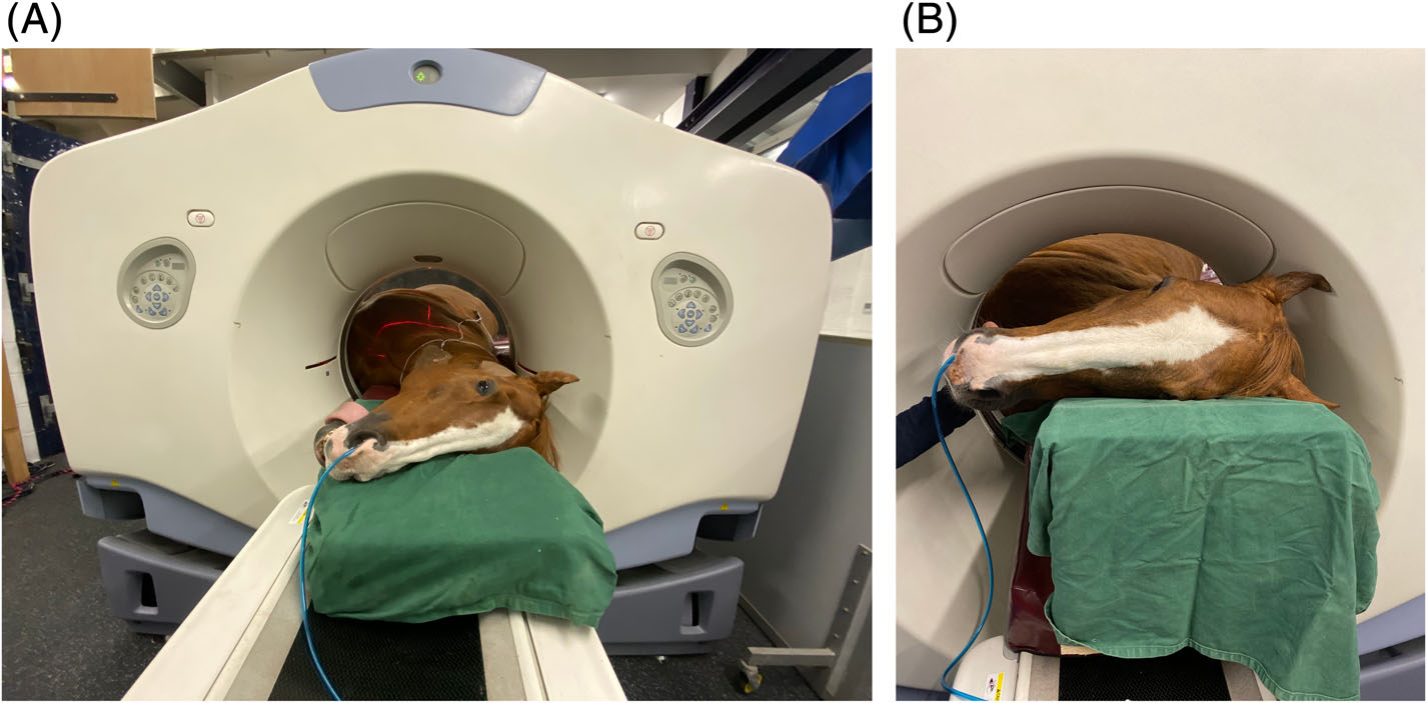
AB, Photographs demonstrating the head and neck positioning of an adult horse for cervical computed tomographic (CT) examination. In B the head is elevated approximately 30° using a custom wooden block

After induction of general anesthesia horses were placed in left lateral recumbency utilizing a specifically adapted table with a scissor lift placed at a predetermined height to match the human gantry table on a purpose made soft horse pad. Computed tomographic technical variables (GE RT 16 [GE Healthcare], Buckinghamshire, UK) were 120 kVp, 400 mA, slice thickness of 1.25 mm, slice gap of 1.25 mm, field of view of 65 cm, with a matrix size of 512 × 512; images were reconstructed using a bone and soft tissue algorithm. Horses were positioned with the shoulder and cervical region off the edge of the equine table to be confluent with the gantry table (Figure 1A,B). A cushion was positioned under the lower left forelimb to prevent radial nerve paralysis. The forelimbs were extended caudally and fixed in this position with ropes that passed through the hindlimbs and were secured to metal hooks in the table. This positioning enabled image acquisition from the brain to the 7th cervical vertebrae in most horses in 1 continuous study, and the third thoracic vertebrae in some horses. Caudal extent of scanning was most dependent on optimal horse positioning and the thickness of the horse from sternum to withers obstructing the horse fitting within the CT gantry.

During acquisition of the plain CT, the head and neck were positioned flat and slightly extended on a soft pad. For cerebrospinal fluid (CSF) removal and subsequent contrast administration, the horses' head and neck were elevated to a 30 to 45° angle using a custom made wooden triangular block. The previously clipped area was aseptically prepared and a sterile iodinated self‐adhesive drape applied to the area.

Puncture of the subarachnoid space at the atlanto‐occipital joint was done in accordance with reported techniques.^13^ This involved maintaining the head in a flexed position with the median axis of the head at a right angle to the median axis of the cervical vertebrae. A sterile 8.9 cm 18‐gauge spinal needle was advanced (with stylet in place) perpendicular to the skin, parallel to the median axis of the cervical spine and directed toward the mandible of the horse. Once the subarachnoid space was punctured (appreciated with a popping sensation and loss of resistance), the stylet was removed to confirm spontaneous flow of CSF. A 20 cm sterile extension set was placed on the end of the catheter and a 3‐way tap used to withdraw 50 mL of CSF, timed over 150 seconds. Immediately after withdrawal of the CSF 20 mL of iohexol (Omnipaque, GE Healthcare AS, Nycoveien, Norway), a nonionic, iodinated contrast media (300 mg I/mL) was injected into the subarachnoid space followed by a 60 mL of a 50:50 dilution of iohexol (Omnipaque, GE Healthcare AS) with sterile Hartmann's solution (Hartmann's Solution, Animal Care LTD, York, UK) (30 mL Iohexol 300 mg I/mL with 30 mL Hartmann's solution) over a total timed 180 second period; a total of 80 mL of injectate into the subarachnoid space. Using this novel approach, the initial non‐diluted contrast media was “chased” by subsequent diluted contrast media subjectively reducing the ventral pooling effect that occurred with standard protocols. A bolus of CSF was then used solely to flush the remaining contrast in the extension set into to the subarachnoid space and the needle was withdrawn.

The head remained elevated after injection for 5‐minutes before being returned to a horizontal position, after which CT image acquisition was performed from the caudal cervical region to the brain. Subsequently horses were moved into the anesthesia recovery box and a series of laterolateral radiographic projections were obtained in extended and maximally flexed positions of the cranial, mid and caudal cervical regions to complete the dynamic myelographic study. After complete image acquisition the head and neck were maintained in an elevated position for a further 10 minutes to encourage caudal flow of iohexol (Omnipaque, GE Healthcare AS), and the horses were recovered from anesthesia using a rope recovery system in accordance with the hospital anesthetic protocol.

## 3 THE CASE REVIEW

**FIGURE 2:**
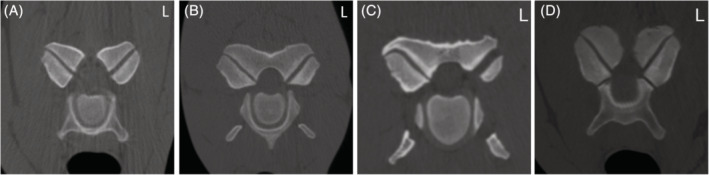
A‐D, Computed tomographic (CT) images depicting grades of degenerative joint disease of the articular process joints. A, Normal (grade 0) articular process joint at C6‐C7 in Case 46; B, grade 1 articular process joint change at C3‐C4 in case 47; C, right sided grade 2 change to the articular process joint of C4‐C5 in case 14; and D, bilateral grade 3 change of the articular process joints with intervertebral foraminae obliteration of C6‐C7 in case 40

**FIGURE 3:**
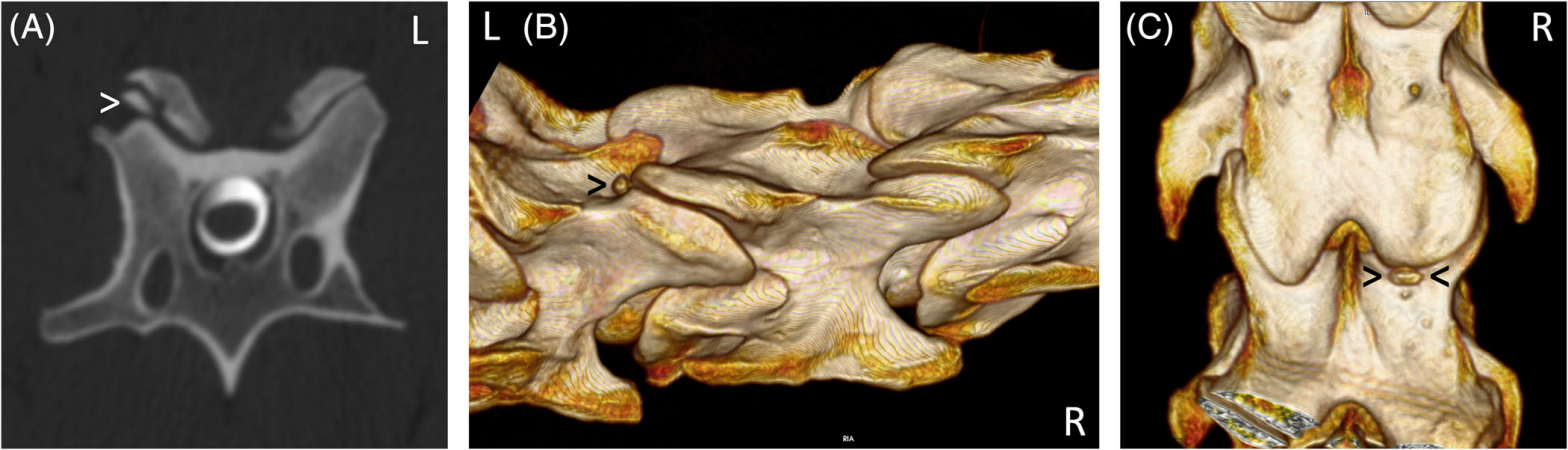
A, Transverse computed tomographic (CT) myelographic image at C5‐C6 in case 5. The white arrowhead indicates an osteochondral fragment associated with the right caudal articular process of C3. B, Right dorsolateral view of a 3D reconstruction with the black arrowhead indicating the osteochondral fragment in the same horse. C, Dorsal view of the same 3D reconstruction with black arrowheads depicting the osteochondral fragment

**FIGURE 4:**
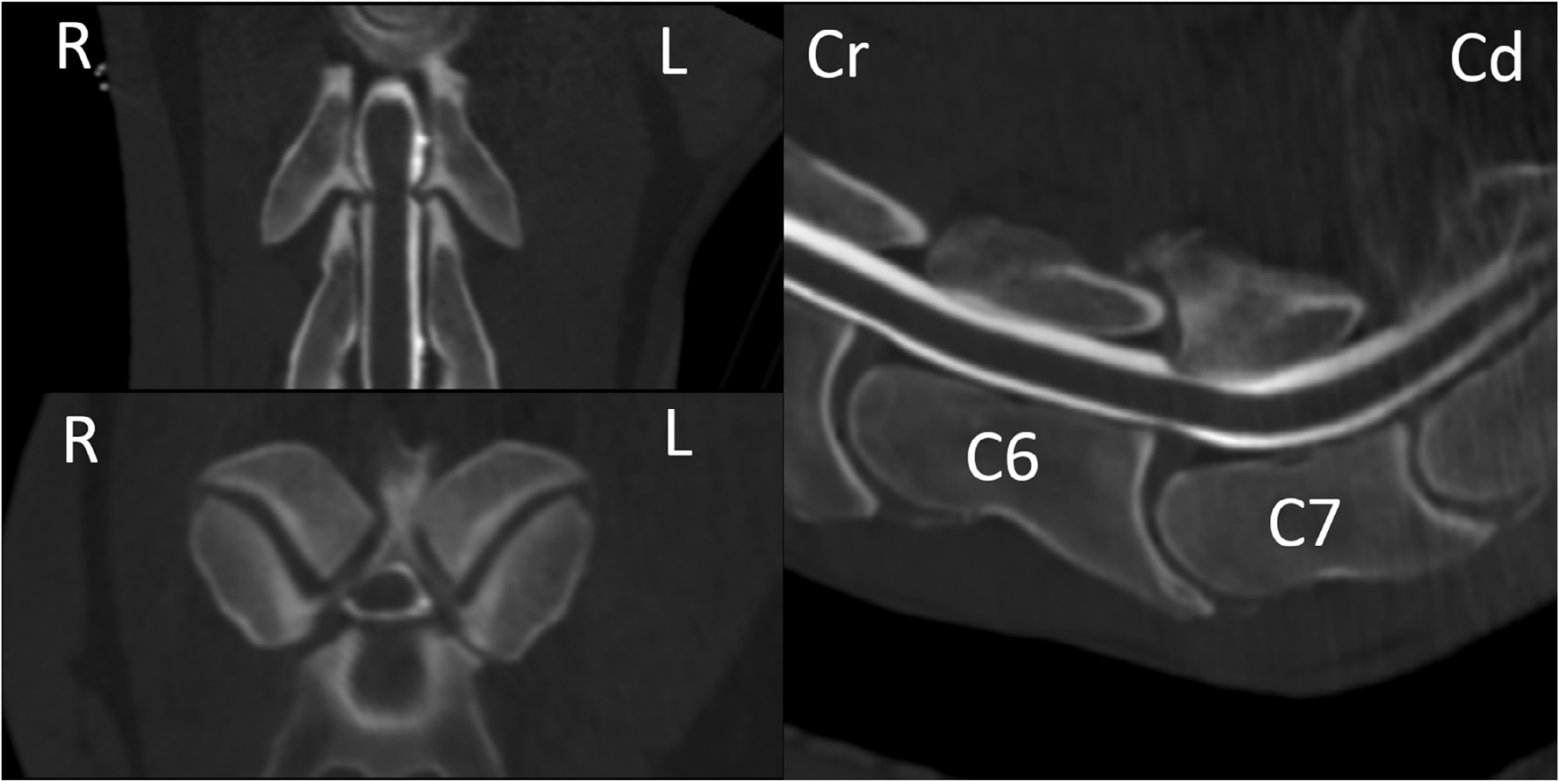
Computed tomographic (CT) myelographic 3D multiplanar reconstruction at the level of C6‐C7 in case 33. Note the profound narrowing of the contrast media column surrounding the spinal cord at this level

**FIGURE 5:**
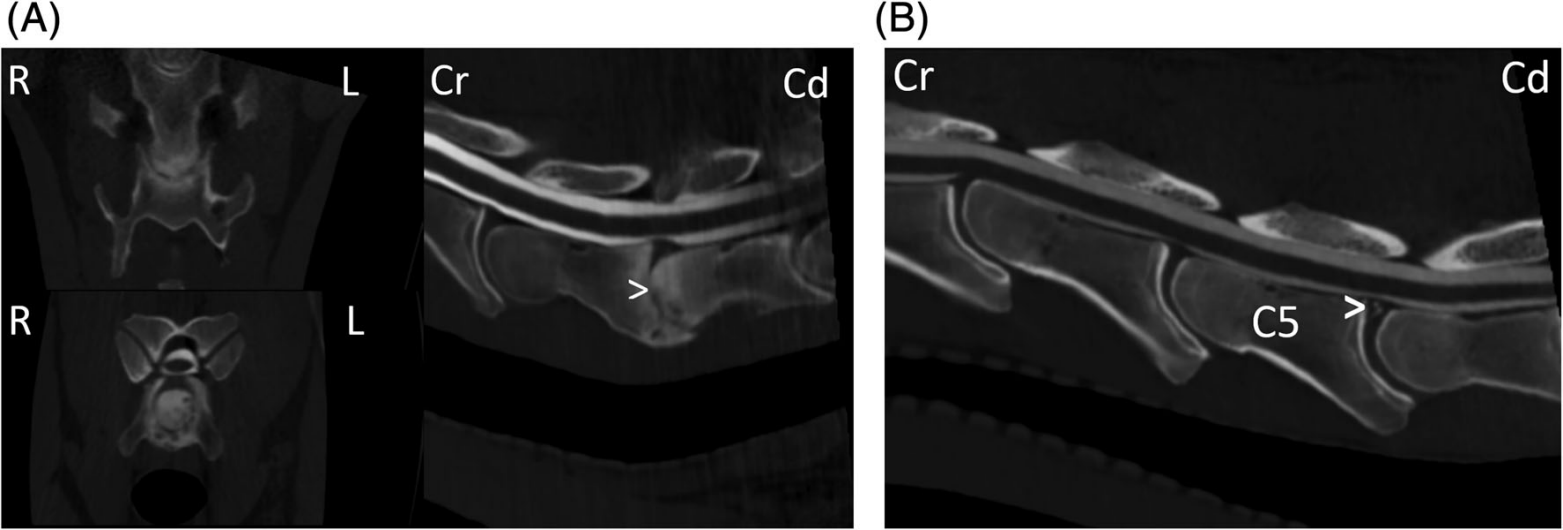
A, Computed tomographic (CT) myelographic 3D multiplanar reconstruction at the level of C6‐C7 in case 16. Note the collapse of the intervertebral disc space (white arrowhead) with severe surrounding increased bone attenuation, and multiple rounded hypoattenuating defects within the vertebral end plates. B, Saggital plane CT myelographic reconstruction at the level of C5‐C6 in case 8. The white arrowhead indicates mineralization within the dorsal portion of the intervertebral disc, not causing spinal cord compression

The presenting clinical signs and findings of neurological examinations of 51 horses that underwent CT myelographic examination and subsequent radiographic myelography (flexed, neutral, and extended views) at Rainbow Equine Hospital for evaluation of the cervical vertebral column were reviewed and recorded. The CT and myelographic images (both CT and radiographic) of all horses were reviewed retrospectively by the authors using a consensus approach. Images were reviewed using a dedicated DICOM viewer (Osirix MD, Pixmeo SARL, Bernex, Switzerland). Each measurement was repeated 3 times and an average recorded. One horse did not have radiographic myelography performed as the CT myelography had provided sufficient diagnostic information in that case. The presence of DJD of the APJ including secondary narrowing or obliteration of the intervertebral foraminae (Figure 2A‐D), osteochondral fragments (Figure 3A‐C), SCC (Figure 4) including the specific location of SCC (dorsoventral, dorsolateral, and circumferential), narrowing, mineralization, or obliteration of the intervertebral disc space (Figure 5A,B), leakage of contrast media from the site of centesis and the presence of bilateral ventral tubercles of C6 and C7 were recorded with a grade of 0‐3 applied to both DJD and SCC (Table 2). Radiographic myelogram findings were considered positive for SCC if there was 50% narrowing of the dorsal and ventral contrast column or 20% narrowing of the dura at any site with the exception of C7‐T1 for which narrowing of 60% of the contrast column or 30% narrowing of the dura or more was used, based on the findings of a recent study by Estell et al.^14^ Anesthetic recovery was graded from 1 to 5 based on previously published scoring systems.^15^ The presenting clinical signs, findings of neurological examination, documented adverse events and short‐term outcome were reviewed retrospectively and have been summarized in Table 1 with respect to the associated CT and myelographic findings. Short‐term outcome was defined as discharge from hospital.

**TABLE 1:** A summary of the presenting clinical signs, neurological examination findings, and perceived clinically significant lesions identified on computed tomographic (CT) myelography and radiographic myelography presented as the percentage of horses with each clinical finding for which a perceived clinically significant lesions was identified on CT myelography and or radiographic myelography

Presenting clinical signs	CT myelographic findings			Radiographic myelography	
	Osteoarthritis	Cord compression	Foramina	Contrast attenuation	Cord compression
Ataxia	79%	21%	50%	36%	7%
General neurological dysfunction	87%	33%	47%	27%	7%
Weakness/collapse	90%	20%	40%	27%	0%
Tripping	78%	11%	78%	11%	0%
Muscle atrophy	100%	0%	0%	0%	0%
Stiffness	81%	44%	63%	25%	6%
Lameness	93%	33%	80%	27%	7%
Not forward	86%	0%	43%	0%	0%
Fever	100%	0%	50%	0%	0%
Neurological examination findings					
Ataxia	100%	62%	75%	38%	0%
Weakness/collapse	89%	32%	71%	32%	11%
Proprioceptive deficits	88%	32%	68%	34%	10%
Reduced range of motion	95%	42%	84%	32%	5%
Muscle wastage	60%	40%	100%	60%	40%
Hyperesthesia/hypoesthesia	79%	36%	64%	36%	0%
Hypermetric gait	87%	43%	83%	43%	17%

**TABLE 2:** Description of the grading scale used to grade degenerative joint disease of the articular process joint (APJ) and spinal cord compression

	Grade 0 (normal)	Grade 1 (mild)	Grade 2 (moderate)	Grade 3 (marked)
Degenerative joint disease	None	Small osteophytes <5 mm on the medial margin	Osteophytes >5 mm with medial projection into the vertebral canal, significant enlargement or remodeling of the APJ, irregularity and lucency of the bone	Subchondral bone erosion, cyst like lesions, extensive sclerosis, osteophytes projecting medially within the vertebral canal, overhanding lateral lesions, significant narrowing
Spinal cord compression	None	Indentation of the contrast column present	Reduction of the contrast column that is >50%	Attenuation of the contrast column with no contrast visible between the bone and spinal cord

## 4 RESULTS

Fifty‐one cases were reviewed. Of these, 17 were mares and 34 were geldings. The median age of horses was 7 years with a range of 1 to 26 years, although the age was unknown in 9 horses. Breeds included Warmbloods and their crosses (n = 20), Thoroughbreds (n = 2), Ponies (n = 3), Irish Draught and their crosses (n = 5), Arabian and their crosses (n = 3), and other breeds (n = 18). The clinical presentations ranged from neurological dysfunction, weakness, stiffness, and thoracic or pelvic limb lameness that was unable to be localized to the limb with diagnostic anesthesia. The findings of the neurological examinations ranged from mild delay in proprioceptive reflexes to marked spinal ataxia and collapse. A summary of the clinical presentation and neurological examinations are detailed in Table 1.

### 4.1 CT and CT myelogram findings

Fifty cases had CT, CT myelography, and radiographic myelography performed, and 1 case had only CT and CT myelography performed as radiographic examination was deemed unnecessary. The CT study incorporated the cervical vertebral column from the brain to the level of C6 in 51 horses with inclusion of the articulation of C6‐C7 in 44/51 horses, C7‐T1 in 34/51 horses, T1‐T2 in 11/51 horses, and T2‐T3 in 4/51 horses. Degenerative joint disease of the APJ was recorded in 1 or more joint in 50/51 horses, of which 44/51 had a site of grade 2 or greater. On CT myelography, SCC was observed in 31/51 horses (61%), of which 16/51 were grade 2 or greater. Of the horses with SCC, 25/31 (81%) horses had lateral or dorsolateral compression, 11/31 (35%) had dorsoventral compression, and 6/31 (19%) had circumferential compression at 1 or more site. Of the 16 horses (52%) considered to have grade 2 or 3 compression, 9/16 (56%) had lateral or dorsolateral compression, 2/16 (13%) had dorsoventral compression, and 6/16 (38%) had circumferential compression at 1 or more site. On radiographic myelography 9/50 horses (12 sites total) had evidence of DCC and ventral contrast column attenuation and 4/50 horses (4 sites total) had evidence of obliteration of the DCC with subsequent impingement of the spinal cord itself, totaling 16 sites. Of the 51 intervertebral junctions with SCC on CT, the number also identified on radiographic myelography (agreement) was 2/7 for grade 1 dorsoventral compression, 2/24 for grade 1 lateral compression, 2/2 grade 2 or 3 dorsoventral compression, 1/11 grade 2 or 3 lateral compression, and 2/7 grade 2 or 3 circumferential compression. There were 6 horses and for which radiographic myelography suggested an additional site of SCC that was not identified on CT myelography, totaling 8 sites, of which 4/8 were located at C4‐C5, 2/8 at C6‐C7, and 2/8 at C7‐T1.

**FIGURE 6:**
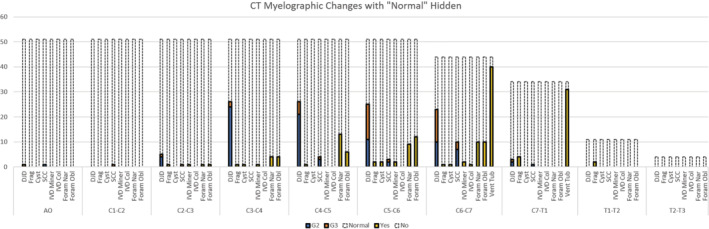
The computed tomographic (CT) myelographic findings and scores (where applicable) for each cervical vertebral joint. Grade 0 = normal and grade 1 = mild; these have been combined together and depicted by the dotted line with no in‐fill; grade 2 = moderate; grade 3 = marked are depicted by color in‐fill. Cyst, cyst within the bone; DJD, degenerative joint disease; Foram Nar, narrowing of the intervertebral foramina; Foram Obl, obliteration of the intervertebral foramina; Frag, fragment of bone; IVD Col, collapse of the intervertebral disc; IVD Miner, mineralization of the intervertebral disc; SCC, spinal cord compression; Vet Tub, presence of the ventral tubercle. The x‐axis pertains to the number of horses

Thirty‐three horses showed narrowing of the intervertebral foramina, obliteration of the intervertebral foramina, or both at 1 or more sites on CT, of which 14/33 had only narrowing. Changes to the intervertebral discs included mineralization in 3/51 of which 1 included bridging bone at 2 sites; collapse and narrowing of the intervertebral disc was observed in 1 horse each. Osteochondral fragments were noted in 11/51 horses and ranged in size from 0.2 cm × 0.3 cm × 0.2 cm to 2 cm × 1.4 cm × 1.2 cm. Osseous cyst‐like lesions were seen in 4/51 horses and a soft tissue mass with intralesional mineralization was observed in 1 horse. A ventral tubercle of C6 and or C7 was unilaterally absent in 4/51 horses. This was not considered clinically relevant. All abnormal findings, the location of the abnormality, the respective grade where appropriate, and site are shown in Figure 6. Additional findings included fragmentation of the spinous process of C7 in 1 horse, subtle leakage of contrast from the subarachnoid space in 35/51 horses after contrast administration (considered incidental and not clinically relevant), mineralization of the ligament of the dens in 3/51 horses, dystrophic mineralization of the lateral neck musculature in 3/51 horses, remodeling of the dorsal arch of C2 and incomplete ossification of the dorsal arch of C1 in 1 horse each, and intra‐articular gas (vacuum phenomenon) in 2 horses.

### 4.2 Outcome

Forty‐seven horses recovered from general anesthesia. Abnormalities after the procedure were recorded in 17/47 horses and included dull demeanor (8/47), fever (3/47), temporary reduced range of movement of the neck (4/47), and temporary worsening of ataxia (7/47). Catastrophic fracture of the carpal bones in 1 limb occurred during anesthetic recovery in 1 horse, necessitating immediate euthanasia. The anesthetic recovery grade was recorded in 42/47 horses. Of these, 25 were graded 5/5, 10 were graded 4/5, 5 were graded 3/5, and 1 each were graded 2/5 and 1/5, respectively. From the recovered 46 horses, 21 had intra‐articular medication of the APJ, 4 underwent cervical stabilization surgery, 6 were subjected to euthanasia, and the remainder were managed conservatively with anti‐inflammatory medication (phenylbutazone [Equipalazone, Dechra LTD, Skipton, UK] or prednisolone [Prednisolone Tablets B.P., Millpledge Veterinary], Retford Notts, UK) and vitamin E supplementation (Veterinary Vitamin E, Farm and Stable, Horndean, UK), in some cases with a recommendation to medicate affected APJ later. The reasons for euthanasia included poor prognosis for athletic soundness, owner preferences, and welfare of the animal.

## 5 DISCUSSION

This study describes a technique for image acquisition and findings of 51 cases of CT myelography extending to the caudal cervical vertebral canal in 1 referral hospital. Image reconstruction techniques of CT examinations allow thorough evaluation of osseous and soft tissues in multiple planes, enabling identification of potential sites of compression circumferentially compared with radiography in which evaluation is typically limited to a sagittal plane.^1^, ^6^, ^7^, ^11^ In a study of 306 ataxic horses, cervical radiography was unreliable, with an accuracy of 40% for diagnosis of SCC when compared with radiographic myelography.^8^ However, even radiographic myelography is limited to evaluation of dorsoventral compression, with lateralized compression requiring dorsoventral radiographic projections which provide inadequate tissue contrast and detail.^8^ In that study, SCC was diagnosed when there was more than 50% reduction in both the dorsal and ventral contrast column, which supports other reports that a 50% reduction in contrast column dorsally or ventrally is not a reliable indicator for compressive lesions and should be interpreted in line with both intervertebral and intravertebral sagittal ratios to improve the accuracy.^3^, ^6^ In addition, site and position (neutral compared with flexed or extended) specific cutoff values have been suggested,^16^ and are appropriate for the cervico‐thoracic articulation.^14^ The finding of more than 50% reduction in DCC at C7‐T1 in normal horses has prompted the suggestion that the cutoff at this site should be increased to more than 60% reduction in the DCC, or more than 20% narrowing of the dural diameter.^14^ In addition, the inherent alterations in the sensitivity and specificity of each measurement during flexion compared with a neutral position at different sites needs to be considered when interpreting the results of radiographic myelography, in particular the increase in false positive diagnoses during flexion at mid‐cervical sites when using a cutoff of values of 50% reduction in DCC or 20% narrowing of the dural diameter, and at C6‐C7 in neutral positioning using these same values.^16^ Vertebral canal cross‐sectional measurements as determined by magnetic resonance imaging (MRI) has been proposed as a more sensitive measure of SCC compared with measurement of vertebral canal height on cervical radiographs.^1^ This volumetric approach allows for variation in vertebral canal shape that still provides adequate space for the spinal cord despite dorsoventral narrowing, and has been shown to correlate well with necropsy findings and histological evidence of SCC.^1^ The finding of an increased frequency of false‐positive diagnoses (by as much as 27%) during flexion in the mid‐cervical region^16^ would also support this theory. This might also explain why only 50% of horses with myelographic evidence of SCC had lesions at necropsy.^8^

Cervical vertebral canal stenosis or cervical vertebral stenotic myelopathy (CVSM) can result in SCC and associated damage to upper motor neurons resulting in spinal ataxia and extensor paresis,^4^, ^17^, ^18^ in addition to damage to spinocerebellar proprioceptive tracts within the spinal cord that might be more pronounced in the pelvic than thoracic limbs because of the more superficial location within the spinal cord. Associated proprioceptive deficits might manifest as toe dragging secondary to flexor paresis and a stiff or hypometric forelimb gait.^17^ In addition to SCC, DJD to the APJs might result in narrowing or obliteration of the intervertebral foramina and compression of the cervical spinal nerve roots where they exit the vertebral column, causing neck pain, stiffness, and forelimb lameness.^4^, ^18^ While oblique radiographic views arguably provide more accurate radiographic evaluation of the APJs than latero‐lateral views, when compared with CT imaging, the sensitivity for identification of DJD is low, although the specificity is relatively high.^19^ Regardless, further evaluation of the cervical vertebral column can be advantageous in horses presenting for neurological dysfunction that includes ataxia or deficits in proprioception, abnormal head carriage or position, focal muscle wastage, lameness that cannot be localized with diagnostic anesthesia to the pelvic or thoracic limbs and abnormal or occasionally sudden exaggerated behavioral responses, in particular in response to flexion or extension of the neck. Both CT and radiographic myelography can identify compressive cord lesions with a similar accuracy,^11^ however, the tomographic detail obtained with CT imaging highlights the superior diagnostic ability of CT.^11^, ^12^, ^18^ However, using current CT techniques, compression of nerve roots associated with bone modeling of the foramina is only implied by proxy of osseous change in the absence of MRI or necropsy confirmation.^18^

Many compressive lesions are only seen on dynamic radiographic myelography.^8^ Narrowing of the vertebral canal was evident in flexion for cranial cervical lesions and extension for caudal cervical lesions, as determined by narrowing of more than 50% of both the dorsal and ventral contrast column at the same location.^8^ In addition, spinal nerve root impingement can also be dynamic with flexion and extension of the neck resulting in increasing and decreasing intervertebral foramina diameter respectively.^20^ The inability to acquire images in maximally flexed positions is a limitation of CT myelography currently, hence the CT examination is supplemented with flexed and extended radiographic myelography images. The size of the horse might limit the caudal extent of vertebral imaging, although with the setup described even large horses with thick necks have had acquisition of images to the level of C7/T1. The author's subjective impression is that with increasing experience of horse positioning, this becomes more straightforward and caudal sites are more readily achieved. The position of the horse on the CT table is critical to maximize the caudal extent of image acquisition. Initial attempts only achieved an imaging field to the level of C5 as the horses were not positioned sufficiently cranial on the patient table. In large horses (approximately >500 kg), the shoulders and sternum can obstruct entry of the caudal cervical region into the CT bore if the horse is not positioned correctly, and in addition, in horses over 220 kg (weight limit for the patient CT table), the CT horse table contacts the CT bore, preventing entry of the caudal cervical vertebrae into the imaging field. With further refinement of the positioning, and development of a customized triangular pad to support the weight of the neck to facilitate such a cranial position on the table, CT imaging to the level of C7 and in many cases to the level of T1 or more caudally is consistently achievable.

The technique used for contrast administration in this study is novel. There is a natural “sump” created at the level of C6‐T1 with the horse in lateral recumbency and the neck and head elevated. During initial cases, this resulted in a relative pooling of contrast material on the dependent sides of the spinal cord and unequal distribution of contrast media surrounding the spinal cord, limiting diagnostic capability. Administration of an initial 20 mL of undiluted contrast media followed immediately by 60 mL of a 50:50 dilution of contrast and saline resulted in more consistent distribution of media circumferentially readily extending to the thoracic vertebrae. The authors consider this approach beneficial to facilitate even contrast distribution without exceeding recommended volumes and concentration of iohexol.

The CT and CT myelographic findings in the cases reported here showed evidence of APJ remodeling at 1 or more joint in 50/51 cases. Although SCC or spinal nerve root compression was a differential diagnosis in all cases, the authors recommend cautious interpretation of subjectively mild bone remodeling of the APJs, and we would suggest that grade 1 lesions might not be clinically relevant in all cases. It is likely that mild osseous remodeling without impingement on adjacent structures is not associated with notable discomfort or clinical signs, and likewise, mild indentation of the subarachnoid space without spinal cord indentation is unlikely to result in true SCC as the area of the vertebral canal might still be sufficient.^1^, ^21^ Grade 2 and grade 3 lesions as defined in this study might be more likely to represent clinically relevant lesions. An approximately 2 mm extension of the medial joint capsule into the vertebral column in normal horses has been identified and it has been suggested that although synovial effusion of the normal APJ alone might not be sufficient to result in SCC, joint effusion in conjunction with osseous and or soft tissue changes might cause SCC.^21^ Although synovial folds and joint effusion are not readily visible on CT, and contrast arthrography was not utilized in this study, the presence of DJD indicates lesions which might result in some degree of synovial inflammation, hence we might be underestimating disease using this premise.

The majority of grades 2 and 3 lesions were localized to C4‐C5, C5‐C6, and C6‐C7, with grade 3 or severe lesions primarily localized to C5‐C6 and C6‐C7, consistent with previously reported findings.^7^, ^8^, ^22^ Although CVSM is frequency associated with lesions at C3‐C4, the primary cause of SCC in the current study was DJD of the APJs, which is similar to other reports^11^, ^22^ and consistent with the signalment of the horses seen at this hospital. Sixty‐one percent of horses in this study showed some degree of SCC, with 81, 35, 19, and 10% of these showing lateral or dorsolateral, dorsoventral, and/or circumferential compression at 1 or more sites respectively. Fifty‐two percent of these were considered to be clinically relevant, of which 56, 13, and 38% had lateral, dorsoventral, and/or circumferential compression respectively at 1 or more sites. These findings are similar to a large study of 306 ataxic horses in which 58% had compressive lesions, although the severity of compression was not reported.^8^ That study also reported that 29% of horses had multiple sites of compression, which is similar to the 26% reported here, albeit only 4% had perceived clinical relevance at multiple sites. The agreement between CT myelography and radiographic myelography in this study was lowest for lateral and circumferential compression identified on CT with only 2/24 grade 1 lateral; 1/11 grade 2 or 3 lateral and 2/7 grade 2 or 3 circumferential lesions showing radiographic contrast attenuation. In comparison, the agreement between CT myelography and radiographic myelography for dorsoventral compression was 2/7 for grade 1 lesions and 2/2 for grade 2 or 3 lesions. Without the addition of histology, it is difficult to surmise whether this disparity is because of overdiagnosis with CT myelography or poor sensitivity of radiographic myelography. Comparatively there were relatively small numbers of horses for which an additional site of SCC was suspected radiographically but not seen on CT myelography, and of these 50% were located at C4‐C5 which would support the presence of dynamic lesions, in agreement with the findings of other authors in which 22% of dynamic lesions were located at this site.^7^ This would further highlight the need for radiographic myelography to complement the CT myelographic findings until CT myelography can be performed in an adequately flexed position, likely requiring a much larger CT gantry size. Alternatively, it might simply reflect the increased occurrence of false‐positive diagnoses at mid‐cervical sites during flexion.^16^

The superior utility of MRI for diagnosis of SCC compared with cervical radiography has been described, largely attributed to the ability to evaluate the vertebral canal tomographically.^1^ A similar comparison between CT and radiography including histological analysis has yet to be reported. However, CT provides some similar 3D advantages. Lateral SCC was identified in 70% of horses with 1 or more sites of compression in this study, supporting the suggestion that encroachment into the vertebral column of the medial pouch of the APJ might occur in a dorsolateral direction, hence the requirement for evaluation of lateral compression.^21^ Although flexed and extended views accentuate dorsoventral compression, the inability to perform laterally flexed views to accentuate lateral compression means the severity of compression might be underestimated with all diagnostic modalities. Currently the need for flexed and extended radiographic views to infer clinical relevance, particularly given the current lack of established measurements for CT myelography, means that radiographic myelography is a necessary adjunct to CT myelography.

The risk of general anesthesia in horses with neurological dysfunction or a history of trauma should be considered. General anesthesia in horses with severe neurological dysfunction that have not been observed to lay down/stand up should be approached with caution, and survey radiography is indicated to identify the presence of fractures or subluxations that might further complicate recovery from anesthesia or require surgical interventions. Adverse effects of intrathecal contrast media administration are rarely reported in horses.^23^, ^24^ The authors have found this procedure to be relatively safe with an incidence of anesthetic recovery^25^ and postmyelography^23^, ^24^ complications comparable to the literature. An incidence of adverse reactions (of any severity) of approximately one‐third of cases, with reactions necessitating euthanasia occurring in 2% of horses has been reported.^24^ Neurological adverse reactions including seizures, worsening of neurologic grade, somnolence, head shaking, and hyperesthesia were noted most frequently in that study, which is consistent with the literature.^23^, ^24^ The incidence of complications was similar in this study, with 5% of cases having a grade 1 or 2 recovery and total morbidity after the procedure of 36% with none showing clinical signs necessitating euthanasia and no horses displaying seizure like activity, hyperesthesia or head shaking. One horse sustained a catastrophic fracture during recovery from general anesthesia making an incidence of 2% in a relatively small sample size, which is similar to the incidence of this occurrence for horses recovering from general anesthesia for any purpose.^25^ Intrathecal administration of iohexol is associated with mild irritation and extradural oedema, often resulting in CSF leucocytosis, although it has been reported that the use of a 300 mg I/mL as used in this study was associated with less irritation than a 350 mg I/mL solution.^23^, ^26^ Increased speed of contrast delivery, increased volume of contrast media delivered, and increased total anesthesia time have been associated with increased risk of adverse neurological events.^24^ However, given the often compromised nature of these horses' proprioception, the incidence of complications being similar to the literature for normal horses supports the relative safety of myelography. This might be in part because of the relatively short anesthetic duration, use of a rope recovery system in horses deemed to be high risk, use of total IV anesthesia and the administration of anti‐inflammatories preoperatively. In the authors' opinion, the risks of anesthetic recovery are outweighed by the superior diagnostic and prognostic information provided by CT and CT myelography.

## CONFLICT OF INTEREST DECLARATION

Authors declare no conflict of interest.

## OFF‐LABEL ANTIMICROBIAL DECLARATION

Authors declare no off‐label use of antimicrobials.

## INSTITUTIONAL ANIMAL CARE AND USE COMMITTEE (IACUC) OR OTHER APPROVAL DECLARATION

Authors declare no IACUC or other approval was needed.

## HUMAN ETHICS APPROVAL DECLARATION

Authors declare human ethics approval was not needed for this study.
